# Use of Computed Tomography Pulmonary Angiography in Emergency Departments: A Literature Review

**DOI:** 10.3390/healthcare10050753

**Published:** 2022-04-19

**Authors:** Lauren E. Thurlow, Pieter J. Van Dam, Sarah J. Prior, Viet Tran

**Affiliations:** 1 Tasmanian School of Medicine, College of Health and Medicine, University of Tasmania, Hobart, TAS 7000, Australia; v.tran@utas.edu.au; 2 School of Nursing, College of Health and Medicine, University of Tasmania, Burnie, TAS 7320, Australia; pieter.vandam@utas.edu.au; 3 Tasmanian School of Medicine, College of Health and Medicine, University of Tasmania, Burnie, TAS 7320, Australia; sarah.prior@utas.edu.au; 4 Emergency Department, Royal Hobart Hospital, Hobart, TAS 7000, Australia; 5 Menzies Institute for Medical Research, University of Tasmania, Hobart, TAS 7000, Australia

**Keywords:** computed tomography angiography, emergency service, hospital, medical overuse, pulmonary embolism

## Abstract

Computed tomography pulmonary angiography (CTPA) has become the most widely used technique for diagnosis or exclusion of a pulmonary embolism (PE). It has been suggested that overuse of this imaging type may be prevalent, especially in emergency departments (EDs). The purpose of this literature review was to explore the use of CTPAs in EDs worldwide. A review following PRISMA guidelines was completed, with research published between September 2010 and August 2020 included. Five key topics emerged: use of CTPAs; explanations for overuse; use of D-dimer; variability in ordering practices between clinicians; and strategies to reduce overuse. This review found that CTPAs continue to be overused in EDs, leading to superfluous risks to patients. Published studies identify that while clinical practice guidelines (CPGs) have a strong effect on reducing unnecessary CTPAs with no significantly increased risk of missed diagnosis, the adoption of these tools by ED clinicians has remained low. This literature review highlights the need for further research into why CTPAs continue to be overused within EDs and why clinicians are hesitant to use CPGs in the clinical setting. Moreover, investigations into other potential strategies that may combat the overuse of this diagnostic tool are essential to reduce potential harm.

## 1. Background

A pulmonary embolism (PE) is an obstruction in one or more of the pulmonary arteries, leading to decreased forward-flow of blood, and is potentially life-threatening. The signs and symptoms of PE vary immensely, from being completely asymptomatic and only found incidentally or on post mortem, to those already experiencing cardiogenic shock—this means that PEs are notoriously difficult to diagnose [1]. In current practice, a key diagnostic tool in identifying PEs is the use of computed tomography pulmonary angiograms (CTPA). Acute PE in CTPAs presents as a filling defect, which can be down to the level of the subsegmental pulmonary artery. The severity can be further assessed through looking for radiological features of right ventricular strain. CTPAs have become the most widely used technique for diagnosis or exclusion of a PE [2], as they provide rapid and reliable results. However, despite the benefits, CTPAs are not without risk. CTPA use has been associated with increased lifetime malignancy risk from exposure to ionising radiation [3], overdiagnosis of clinically inconsequential PEs [4] and contrast-related complications, as well as contributing to the growing costs of healthcare [5]. Other uncommon but significant consequences of contrast-enhanced imaging, such as CTPAs, include extravasation of contrast and anaphylaxis [6]. The emergency department (ED) has been singled out as an area within hospitals where overuse of this imaging may be significant [2], potentially contributing to patient harm and rising costs.

Clinical practice guidelines (CPGs) aid clinicians in identifying patients in which PE can be safely excluded, without the use of a CTPA. Some prominent examples of CPGs include the Wells score, the Geneva score, the Charlotte rule, and their associated simplified versions. A negative D-dimer result can exclude PE in low-risk patients; however, the D-dimer can sometimes result in a false positive, especially in older patients. As a result, the age-adjusted D-dimer cut-off point was developed in order to increase specificity of the test in older populations [7]. For some low-risk patients, the PE Rule-Out Criteria (PERC) can be used to rule out PE without any diagnostic testing. PERC is a validated clinical decision rule that is just as sensitive and more specific than a D-dimer and can therefore reduce the need for a CTPA [8]. Choosing Wisely Australia is a campaign focused on improving the safety and quality of healthcare by providing recommendations to reduce unnecessary medical interventions. In 2015, they recommended that no diagnostic testing for PE is to be conducted unless indicated by a validated CPG [9].

This literature review aims to firstly explore the current use of CTPAs in EDs worldwide. Secondly, it aims to identify the reasons behind this pattern of use and intends to summarise strategies from the literature to combat any overuse.

## 2. Search Strategy

**Figure 1:**
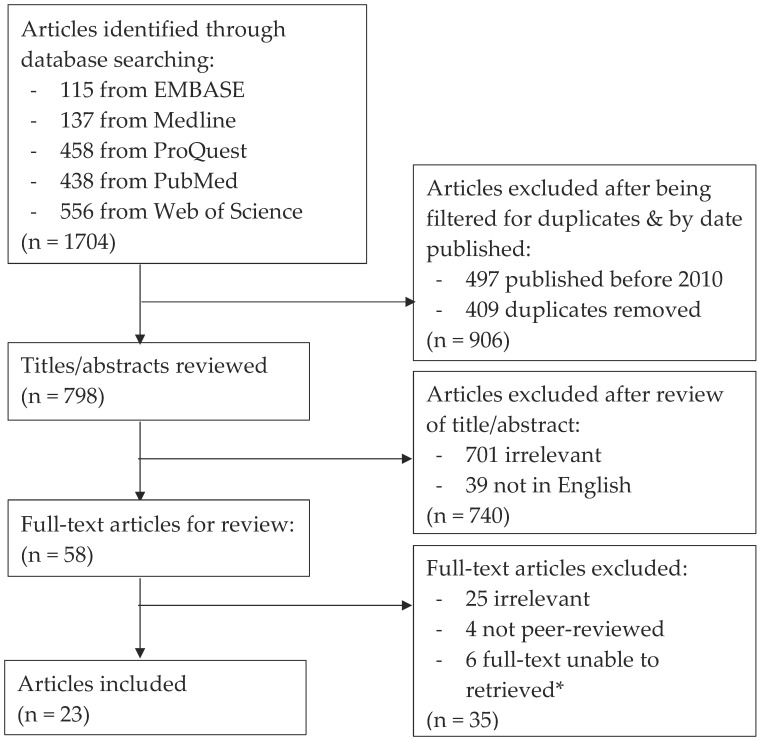
PRISMA research method. Summary of articles identified, screened and included in literature review, adapted from Moher et al. [11]. * Articles where the full text could not be retrieved were published as supplement articles (abstracts presented at a conference).

This literature search reviewed studies published in English, in peer-reviewed journals, between September 2010 and August 2020. A systematic search strategy was undertaken following the PRISMA guidelines to ensure the method was structured, transparent and repeatable [10]. The databases EMBASE, Medline, ProQuest, PubMed and Web of Science were searched for eligible articles. Searches used a combination of keywords related to the concepts of overuse, computed tomography pulmonary angiography and emergency departments (Table 1). There was a full analysis of all words included in the titles and abstracts of each article, and those that did not directly relate to the use of CTPAs in EDs were excluded. Those papers deemed potentially relevant were then analysed in full text for eligibility. The flow chart in Figure 1 outlines the articles identified, screened and included in the review.

## 3. Results

Twenty-three studies met the eligibility criteria (Figure 1), the characteristics of which are presented in Appendix A. The majority (20 studies) of the literature originates from the United States and Canada, with just one study each from Australia, Europe and the Middle East. Much of the literature sourced were original research studies that aimed to calculate the CTPA positivity yield in hospital EDs. Some of the studies further analysed the effects of specific interventions on this yield. These findings are also presented in Appendix A. This literature review provides clear evidence and support to the idea that CTPAs continue to be overused in EDs; however, only a small number of studies present explanations for this finding. Further analysis of these studies resulted in the identification of five key topics within the literature. These are: the use of CTPAs; explanations for overuse; the use of D-dimer; variability in ordering practices between clinicians; and strategies to reduce overuse. Each of these key topics is explored in further detail below.

### 3.1. Use of CTPAs

Strong evidence was found indicating that CTPAs are overused in EDs [6,12,13,14,15]. The relationship between increasing CTPA use in EDs and lower diagnostic yields is well established [1], where a low diagnostic yield is often indicative of overuse, as it implies patients are being inappropriately screened for the existence of a PE [13]. Similarly, a high diagnostic yield may be an indicator of underuse, which may lead to missed diagnoses. The ‘ideal’ CTPA positivity rate is unknown and highly debatable; however, through their own review, Mountain et al. [2] nominated 15.3% to be the ideal diagnostic yield for CTPA scans. This review of the literature found that on average, a PE was diagnosed in 11.74% of CTPAs conducted (Appendix A)—a number below their nominated ‘ideal’ yield.

The results suggests that many CTPAs are unnecessary [16], with a 2015 study identifying that 49.5% of CTPAs conducted in the ED were potentially avoidable [15]. Another study identified that 41% of CTPAs were inappropriately ordered—meaning that the patients were scanned against recognised CPGs that had indicated that they had a low pre-test probability of PE, and a D-dimer assay was either not completed or was negative [14].

A CTPA that is not otherwise indicated by a CPG is sometimes undertaken, and subsequently deemed reasonable, through finding an alternative diagnosis that explains the patients’ clinical presentation [17,18]. Anjum et al. [19] found that 10.1% of patients who had a negative CTPA had received an alternative diagnosis from the scan. Similarly, their study also demonstrated that in patients’ undergoing CTPA, incidental findings such as pulmonary nodules are almost as likely as a diagnosis of PE. They found incidental findings on 13.1% of the CTPA scans conducted, and 88.3% of those incidental findings were deemed clinically insignificant by follow-up CT imaging. While not explicitly mentioned in the studies included in this review, it is important to note that in critically ill patients, the absence of PE may be considered a significant finding.

### 3.2. Explanations for Overuse

Limited original research was found on explanations as to why these scans are overused within EDs. A 2012 study conducted through surveys found that a “fear of missing PE” was a motivating factor in over 55% of CTPAs ordered, while the same study identified a “fear of being sued” as the reason for ordering 5% of CTPAs in this study [20]. Only one qualitative study was found in this review, undertaken by Gyftopoulos et al. [21], and they also discussed clinicians’ fears of malpractice and litigation as a motivator for over-ordering CTPAs. Other emergency specialists interviewed simply felt that they needed to order a CTPA for any patient in which the diagnosis of PE could not otherwise be excluded [21].

Some clinicians interviewed by Gyftopoulos et al. [21] indicated that they thought the actual process of ordering a CTPA was just too convenient. One clinician suggested implementing a feature into the electronic ordering system where the clinician must enter the score of a recognised PE screening tool, otherwise the order will not be processed. It was thought that it might make the ordering clinician reassess their decision. This idea has also been recommended in many other studies found [17,22,23]; however, only one study included in this review evaluated the effectiveness of this intervention. Raja et al. [24] incorporated a set of questions, developed from validated CPGs, into their electronic ordering system. These included asking clinicians to select the patients’ D-dimer result and pre-test probability of PE, and there was also the option not to answer the questions. The implementation of this program was associated with a 20.1% decrease in the use of the service, as well as a 69% increase in the CTPA positivity rate.

Booker and Johnson [12] outline the demand for rapid patient turnover in the ED as a barrier to quality improvement in regard to CTPA ordering practices. They suggested that this may lead to ‘blanket ordering’, which “attempts to reach a diagnosis as quickly as possible despite cost and patient safety” [12]. As CTPAs are both the ‘gold standard’ and exceptionally fast, some clinicians expressed the belief that it is better for the patient to have the scan, rather than keep them in the ED for an extended period of time awaiting a D-dimer result [21].

### 3.3. Use of D-Dimer

The use of the D-dimer itself was also debated, with some clinicians in the study by Gyftopoulos et al. [21] expressing that the D-dimer was too widely used, especially in patients with a very low clinical suspicion of PE. This is because despite the known false positivity rate, an elevated D-dimer caused some clinicians to feel forced into ordering a CTPA—even when they thought it was unnecessary [21]. The PERC was formulated in 2004 to reduce the use of unnecessary D-dimer testing [8]. Buchanan et al. [25] found inconsistent adherence to the PERC, with over 25% of PERC-negative patients undergoing further testing for PE. They also found that over 7% of these patients underwent advanced imaging, such as a CTPA, without prior D-dimer testing.

Underutilisation of D-dimer testing leading to unnecessary CTPAs was a common theme found [12,14,15,16,17]. Booker and Johnson [12] discovered that although the literature has validated the D-dimer as a safe way to rule out PE, this has not been embraced in the clinical setting, with 10% of CTPAs ordered on patients with negative D-dimers in low-score patients. Al Dandan et al. [17] also identified a significant underutilization of the D-dimer assay in their study, as it was not used despite recommendation by CPGs, in over 18% of patients. Two separate studies identified that over 55% of patients with a low pre-test probability for PE did not have a D-dimer prior to advanced imaging, such as a CTPA [14,15]. Some studies hypothesised that increased patient load and time constraints within a busy ED may play a role in the underutilisation of D-dimer testing [17,21], while Venkatesh et al. [16] theorised that clinicians may have a preference towards CTPA as it is a more definitive test.

### 3.4. Variability between Clinicians

Senior clinicians tend to have a higher positive scan rate than their junior counterparts [22,26]. Kindermann et al. [22] suggest that this may be because junior clinicians prefer to err on the side of caution and order a CTPA, rather than risk missing a PE. Other studies identified inconsistencies in ordering patterns and diagnostic yield of CTPAs among physicians working in the same clinical environments [1,12,18]. Booker and Johnson [12] suggest that measuring clinician-specific data may provide a better understanding of the variability between diagnostic approaches, while Chen et al. [18] proposed that the use of CPGs could formulate a more standardised approach between clinicians to CTPA use.

### 3.5. Strategies to Reduce Overuse

Through this review of the literature, one strategy emerged as the most prominent in tackling overuse of CTPAs in EDs. This was the implementation and strict adherence to validated CPGs. In an American study conducted by Yan et al. [27], it was found that when clinicians adhered to CPGs, their CTPA positivity yield was nearly double in comparison to when the CPGs were overridden. Despite the evidence supporting the use of CPGs, research suggests they are not widely adopted by ordering physicians [17,20,23,25], with Simon et al. [28] identifying that between 25–37% of scans were not ordered in accordance with CPGs.

Similarly, Crichlow et al. [6] also found CPGs were underutilised in their study. They suggested that a potential barrier to the use of CPGs is that clinicians may feel gestalt is superior. Chen et al. [18] support this and highlight that while gestalt is safe, it is less specific than CPGs and may lead to higher utilisation of CTPAs. They also note that gestalt is much less reliable in junior clinicians. Ferguson et al. [23] found a 250% increase in positive CTPAs when clinicians used CPGs over gestalt. They also found that CPGs were less likely to be adhered to at a tertiary care centre than in a community hospital, despite being staffed by the same physicians. They suggested that this may be due to easier access to a CT scanner at the tertiary care centre, stating “the hypothesis that increased CT availability, not appropriate patient care, drives increased utilisation is a major concern” [23].

## 4. Discussion

Through this review of the literature, five key topics emerged. The first four highlight strong, consistent evidence that CTPAs are still overused [6,12,13,14,15], with limited evidence explaining why this occurs. This is a concerning finding, particularly in the light of the potential adverse events which could occur by using CTPAs. One of the strategies to overcome overuse could be to ensure that patients are well informed about their treatment options. It has been suggested that combining healthcare professionals’ skills in evidence-based medicine with patient’s goals and preferences has the potential for the provision of appropriate treatment [29]. The literature also highlights that the inappropriate use of D-dimer testing contributes further to the overuse of CTPAs, with significant underutilisation of the test evident across multiple studies [12,14,15,16,17]. Some studies included in this review identified variability in ordering practices between junior and senior clinicians [1,12,18]. While this variation could potentially be explained by the fact that senior clinicians are more experienced and more likely to see the critically unwell patients in the department, it also suggests that a standardised approach to assessment of patients with suspected PE is not being widely adopted. A diagnosis is made through context-dependent collaboration of members of the diagnostic team, including patients, senior medical officers and nurses [30]. A standard approach might be achieved by ensuring that the senior members of the diagnostic team work closely together with the more junior members, as is the case in most public emergency departments in Australasia.

Our findings clearly highlight that adherence to CPGs reduces unnecessary CTPAs [6,24,31]. As a result, there have been significant efforts to promote the use of CPGs prior to ordering CTPAs. This is evidenced by the fact that clinicians who utilise CPGs are more likely to have a higher diagnostic yield [20]. Despite this, studies to date suggest that clinician adoption of CPGs is low [17,20,23,25]. This indicates that appropriate utilisation of CPGs requires a multimodal approach to change the current culture of investigating for PEs. Part of this approach might be situated in the concept of changing behaviours. Some medical officers are known to be assessing patients in a certain way for extended periods of time without any issues and therefore the case for change might not be clear [32]. Target education for this group might help in convincing to change. However, the reasons why clinicians may not utilise a CPG are not fully understood as there is limited evidence. Only one study included in this review directly asked clinicians about their clinical workup of patients with suspected PE by adopting a qualitative approach. Many of the other studies included are retrospective quantitative studies, meaning the current available research on this topic is limited in study design. More qualitative studies are needed in order to develop a deeper understanding of the emerging themes, especially with regards to clinician ordering practices.

It is known that CTPAs are not without adverse effects, including exposure to ionising radiation and contrast-related complications. In addition to risks to the patient, CTPAs are also demanding both financially and in the way of clinician time, as unnecessary scans can create delays for other, more urgent scans, as well as removing clinicians from the floor to transport the patient to the scan. Due to these costs, overuse of CTPAs has been identified as an example of low-value care, which has been recognised by the Royal Australian and New Zealand College of Radiologists and the Choosing Wisely campaign [9] and therefore attempts need to be made to reduce or eliminate overuse.

## 5. Limitations

This literature review focused only on manuscripts published in peer-reviewed journals between September 2010 and August 2020. There may be additional unpublished or non-peer-reviewed data, as well as papers published outside of this date range, available that provide further insight into the current use of CTPAs. A majority of the studies included in this review (16 out of 23) are from the United States of America; therefore, applicability to different healthcare settings is difficult to interpret. Additionally, although this review focusses on the clinical factors related to the use of CTPAs, it did not directly explore the actual costs and risks associated with CTPA scans. Lastly, any interpretation of D-dimer results is beyond the scope of this review.

## 6. Implications for Practice

This literature review identified limited original research on explanations as to why CTPAs are overused within EDs. Many authors suggested potential explanations for overuse; however, only one study adopted a qualitative approach and interviewed clinicians to uncover genuine explanations for ordering patterns. For this reason, additional original qualitative research into this particular aspect of the topic is recommended.

The mandatory assignment of the Wells score and/or other validated CPGs prior to ordering a CTPA was discussed in multiple articles [17,21,22,23]; however, the effectiveness of this intervention was not measured. Raja et al. [24] applied a similar intervention with meaningful results; however, they did not use a validated CPG. Further studies into the effectiveness of this intervention are recommended.

## 7. Conclusions

This review found that CTPAs are overused in EDs worldwide, leading to superfluous risks to both patients and medical professionals. Published studies identify that while CPGs have a strong effect on reducing unnecessary CTPAs with no significant increased risk of missed diagnosis, the adoption of these tools by ED clinicians has remained low. This literature review highlights the need for further research into why CTPAs continue to be overused within EDs, why clinicians are hesitant to use CPGs in the clinical setting, and into other potential strategies that may combat the overuse of this diagnostic tool.

## Figures and Tables

**Table 1:** Concept & Keyword Mapping Table.

	Concept 1	Concept 2	Concept 3
Key Concepts *	Overuse	CTPAs	Emergency Department
Free Text/Natural Language Terms	Over-use, over use, over used, use, utili#ation, overutili#ation, yield, diagnostic yield, positivity rate	CTPA, PCTA, CT pulmonary angiograms, CT pulmonary angiogram, CT pulmonary angiography, computed tomography pulmonary angiograms, computed tomography pulmonary angiogram, computed tomography pulmonary angiography	ED, emergency room. ER, accident & emergency, a & e, a&e
Controlled Vocabulary/Subject Terms	“Medical Overuse”	“Computed Tomography Angiography & Pulmonary Artery”	“Emergency Service, Hospital”

* Each term within a concept was linked with the operator ‘OR’, while each concept was linked with the operator ‘AND’.

## Data Availability

Not applicable.

## Appendix A

**Table A1:** Characteristics of Studies Included in Literature Review.

Author(s)	Year	Country	Study Design	*n*	CTPA Positivity Yield Pre-Intervention	Intervention (If Applicable)	CTPA Positivity Yield Post-Intervention	Potentially Avoidable CTPAs
Al Dandan et al.	2020	Saudi Arabia	Retrospective Observational Study	353	–	–	18.7%	–
Anjum et al.	2019	Canada	Retrospective Cohort Study	1708	–	–	13.6%	–
Booker et al.	2017	United States of America	Retrospective Review	412	8.7%	Clinician Education	9.2%	–
Buchanan et al.	2017	United States of America	Prospective Observational Study	3024	–	–	–	–
Chen et al.	2015	Canada	Cross-Sectional Retrospective Study	835	–	–	17.8%	–
Crichlow et al.	2012	United States of America	Prospective Cohort Study	152	–	–	11.8%	9.2%
Ferguson, Low & Fung	2019	Canada	Retrospective Cohort Study	510	5.9%	Clinical Practice Guidelines	15.0%	–
Gyftopoulos et al.	2018	United States of America	Semi-Structured Interviews	33	–	–	–	–
Kanaan et al.	2013	United States of America	Retrospective Review	200	8.0%	Clinician Education	10.0%	–
Kindermann et al.	2014	United States of America	Retrospective Review	12,883 ^1,2^	–	–	5.9% *	–
Kline et al.	2020	United States of America	Retrospective Review	42,267 ^1^	–	–	3.0% ^	–
Mountain et al.	2016	Australia	Retrospective Review	7077	–	–	14.6%	–
Osman et al.	2018	United States of America	Retrospective Review	295	–	–	5.4%	–
Parikh et al.	2015	United States of America	Retrospective Review	196	–	–	10.7%	–
Perelas et al.	2015	United States of America	Retrospective Review	646	–	–	9.4%	49.5%
Raja et al.	2012	United States of America	Retrospective Review	6838	5.8%	Clinical Practice Guidelines	9.8%	–
Rohacek et al.	2012	Switzerland	Prospective Cohort Study including a Survey	328	14.5%	Clinical Practice Guidelines Pre-Ordering Questionnaire	19.2%	–
Salehi et al.	2020	Canada	Retrospective Observational Study	2788	–	–	10.3%	–
Shujaat, Shapiro & Eden	2013	United States of America	Retrospective Review	231	–	–	20.7%	–
Simon et al.	2019	United States of America	Retrospective Review	212	–	–	8.5%	8.7%
Stojanovska et al.	2015	United States of America	Prospective Cohort Study	602	–	–	10.0%	–
Venkatesh et al.	2012	United States of America	Prospective Observational Study	5940	–	–	–	32%
Yan et al.	2017	United States of America	Retrospective Cohort Study	2993	4.2%	Clinical Practice Guidelines	11.2%	19.7%
**Average CTPA Positivity Yield ^3^**							**11.74%**	

^1^ This result has been calculated from the information presented within the study. ^2^ This result is inclusive of a small number of V/Q scans. ^3^ Post-intervention rate used where possible.
